# Typhus Fever

**Published:** 1856-05

**Authors:** Edwin R. Maxson

**Affiliations:** Geneva, N. Y.


ART. II.—*Typhus Fever.* By EDWIN R. MAXSON, M. D., Geneva, N. Y.

Typhus fever, whether produced by filth, confined air, or a peculiar virus, or morbific agent generated in, and exhaled from, the bodies of those suffering from the disease, is characterized by a train of symptoms indicative of prostration of the cerebro-spinal system; and also, of the sanguiferous. And after an irritated reäction of the sanguiferous system, of longer or shorter duration, there is a general collapse, or sinking of all the powers of the system, in many cases at least.

Now let us suppose, as is probable, that this virus, or morbific agent, is received into the blood through the mouth, with the saliva, and also, through the membranes of the air-cells, and absorbent lymphatic vessels of the lungs. In either case it soon reaches the circulation, and passes with the blood to every part of the system, acting more or less injuriously on every part, the capillaries of which this virus is made to traverse.

It is easy to see, that while every part of the system may suffer from the direct effects of this agent acting upon the capillary vessels, and perhaps also upon the nerves, supplying the inner surface of the heart, and the larger arteries and veins, the principal effect is upon the cerebro-spinal system, thereby deranging through the nerves the functions of the different organs of the body.

And though we cannot tell exactly the manner in which the virus operates through the cerebro-spinal and nervous systems, it is evident, from the symptoms which mark the stage of invasion of typhus, that the first impression of this morbific agent upon the brain, is of a debilitating character; gradually rendering the brain incompetent to generate sufficient nervous influence, or vital force, to keep up a healthy action of the various organs.

Hence, the loss of appetite, giddiness, nausea, dejected countenance, and general disinclination to mental and corporeal action, which so generally occur during the incipient stages of typhus; symptoms which we should naturally suppose would occur from a debilitating agent, acting upon the cerebro-spinal and nervous system, impairing the generation and distribution of the nervous influence.

This derangement appears, at first, to involve mainly the functions of parts supplied more directly by the cerebro-spinal nerves, including, of course, those supplied by the pneumogastric. But sooner or later, the vital or involuntary functions become impaired, probably from inability on the part of the brain to supply the sympathetic nerve with sufficient vital energy; and, as a consequence, the action of the heart and arteries becomes weak, the blood recedes from the extremities and surface of the body, and a chill, or chills, is the result.

During the continuance of this chilly stage, the brain suffers more or less from congestion, as well as all the internal viscera, and, especially, the alimentary mucous membrane. And, as this congestion continues, it produces an irritated condition of the cerebro-spinal system, and through that, of the sanguiferous, developing the symptoms which mark the stage of excitement.

Now this stage of excitement in which there is an imperfect effort at reäction, and that of an irritated character, is sooner or later followed, either by a more perfect and healthy reäction, and slow convalescence, the various functions of the body being gradually restored; or else, by a collapse which indicates an inability, on the part of the system, to keep up even an irritated action; and which too often terminates in an entire suspension of vitality.

What, then, are the indications in the treatment of typhus?

Shall we bleed, purge, and starve our patients, and thus increase the debility which the morbific agent has produced? Or shall we strive to support the sinking energies of the system, and thus enable it to bear up under and throw off, with its various excretions, the debilitating morbific agent it has so unfortunately imbibed.

From my experience in the treatment of typhus fever, for the past few years, I am compelled to believe, that with a proper sustaining course of treatment, nearly every case of typhus may be arrested, and the patient convalescent, by the fifth or sixth day; and that very few need be kept in longer than the ninth day, if attended to in season, and before any serious local inflammation has taken place.

The indications then are, to remove from the alimentary canal, any irritating matter which it may contain; to equalize the circulation; to promote perspiration; and to support the sinking powers of the system by tonics and a due amount of proper nourishment.

I therefore usually give, at first, two or three blue pills, and follow with half an ounce of castor oil; use warm pediluvia, morning and evening, the first day or two; also rubbing the whole length of the back with a tepid infusion of capsicum in vinegar, and, generally, give the sulphate of quinine and Dover's powders, of each, grs. iv, every six hours, at first, for a day or two. I then discontinue the Dover's and give pulv. camph., gr. i, with quinine sulph., grs. iii, every six hours, and continue till the fever is arrested, giving crust coffee, with milk, at first freely, as nourishment, and as soon as the stomach will retain it, toast every six hours; and, by degrees, other nourishing and digestible food, as the appetite gradually returns and calls for it.

Such are the means which I have found most effectual in arresting typhus fever; and even in cases in which, through neglect, or maltreatment, local inflammation has supervened, I have found this course to do well, in conjunction with sinapisms, dry cupping, or blistering, as the case may require.

